# Tris(1,10-phenanthrolin-1-ium) hexa­cyanidoferrate(III) ethanol monosolvate trihydrate

**DOI:** 10.1107/S1600536812017990

**Published:** 2012-04-28

**Authors:** Xiao-Qing Liu, Deng-Yong Yu, Ai-Hua Yuan

**Affiliations:** a School of Material Science and Engineering, Jiangsu University of Science and Technology, Zhenjiang 212003, People’s Republic of China; b School of Biology and Chemical Engineering, Jiangusu University of Science and Technology, Zhenjiang 212003, People’s Republic of China

## Abstract

The asymmetric unit of the title complex, (C_12_H_9_N_2_)_3_[Fe(CN)_6_]·C_2_H_5_OH·3H_2_O, consists of two half [Fe(CN)_6_]^3−^ anions located on inversion centers, three 1,10-phenanthrolin-1-ium cations, [Hphen]^+^, an ethanol and three water solvent mol­ecules. The average Fe—C and C—N bond lengths are 1.942 (6) and 1.154 (3) Å, respectively, while the Fe—C—N angles deviate slightly from linearity with values ranging from 177.8 (2) to 179.7 (2)°. The Fe^III^ atoms adopt a distorted octa­hedral geometry. All the species are linked through O—H⋯N, N—H⋯O and O—H⋯O hydrogen-bonding inter­actions, resulting in a three-dimensional supra­molecular network.

## Related literature

For general background to hexa­cyanido­metalate(III)-based complexes, see: Andruh *et al.* (2009 ▶); Tokoro & Ohkoshi (2011 ▶). For background to complexes containing hexa­cyanido­metalate and phen ligands, see: Koner *et al.* (2005 ▶).
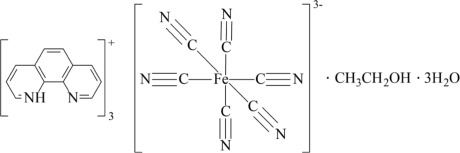


## Experimental

### 

#### Crystal data

(C_12_H_9_N_2_)_3_[Fe(CN)_6_]·C_2_H_6_O·3H_2_O
*M* *_r_* = 855.72
Monoclinic, 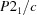

*a* = 20.5744 (18) Å
*b* = 14.8742 (13) Å
*c* = 14.1594 (12) Å
β = 109.123 (1)°
*V* = 4094.1 (6) Å^3^
*Z* = 4
Mo *K*α radiation
μ = 0.43 mm^−1^
*T* = 173 K
0.21 × 0.18 × 0.16 mm

#### Data collection

Bruker APEXII diffractometer
Absorption correction: multi-scan (*SADABS*; Bruker, 2004 ▶) *T*_min_ = 0.915, *T*_max_ = 0.935
35350 measured reflections
9412 independent reflections
6179 reflections with *I* > 2σ(*I*)
*R*_int_ = 0.049

#### Refinement

*R*[*F* ^2^ > 2σ(*F* ^2^)] = 0.042
*wR*(*F* ^2^) = 0.115
*S* = 1.01
9412 reflections
555 parameters
H-atom parameters constrained
Δρ_max_ = 0.42 e Å^−3^
Δρ_min_ = −0.46 e Å^−3^

### 

Data collection: *APEX2* (Bruker, 2004 ▶); cell refinement: *SAINT* (Bruker, 2004 ▶); data reduction: *SAINT*; program(s) used to solve structure: *SHELXS97* (Sheldrick, 2008 ▶); program(s) used to refine structure: *SHELXL97* (Sheldrick, 2008 ▶); molecular graphics: *DIAMOND* (Brandenburg, 2006 ▶); software used to prepare material for publication: *SHELXTL* (Sheldrick, 2008 ▶).

## Supplementary Material

Crystal structure: contains datablock(s) I, global. DOI: 10.1107/S1600536812017990/pv2532sup1.cif

Structure factors: contains datablock(s) I. DOI: 10.1107/S1600536812017990/pv2532Isup2.hkl

Additional supplementary materials:  crystallographic information; 3D view; checkCIF report

## Figures and Tables

**Table 1:** Hydrogen-bond geometry (Å, °)

*D*—H⋯*A*	*D*—H	H⋯*A*	*D*⋯*A*	*D*—H⋯*A*
O1—H1*WA*⋯N1^i^	0.85	1.97	2.822 (2)	176
O1—H1*WB*⋯N2^ii^	0.85	1.89	2.732 (3)	172
O2—H2*WA*⋯N3^iii^	0.85	1.97	2.809 (3)	169
O2—H2*WB*⋯N4^iii^	0.85	1.92	2.761 (3)	171
O3—H3*WA*⋯N5	0.85	2.05	2.891 (3)	168
O3—H3*WB*⋯N6^iv^	0.85	1.93	2.769 (3)	168
N7—H7*A*⋯O1	0.88	1.83	2.681 (2)	161
N9—H9*A*⋯O3	0.88	1.80	2.643 (2)	159
N12—H12*A*⋯O2	0.88	1.84	2.635 (3)	150
O4—H4⋯O1^v^	0.84	1.99	2.813 (3)	167

Symmetry codes: (i) 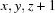; (ii) 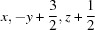; (iii) 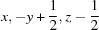; (iv) 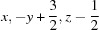; (v) 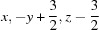.

**Fig. 1.**
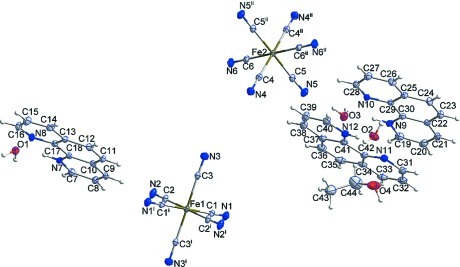
ORTEP diagram of the title complex, showing the 30% probability thermal motion ellipsoid. Symmetry codes: (i) -x + 1, -y + 1, -z + 1; (ii) -x + 2, -y + 1, -z + 1.

**Fig. 2.**
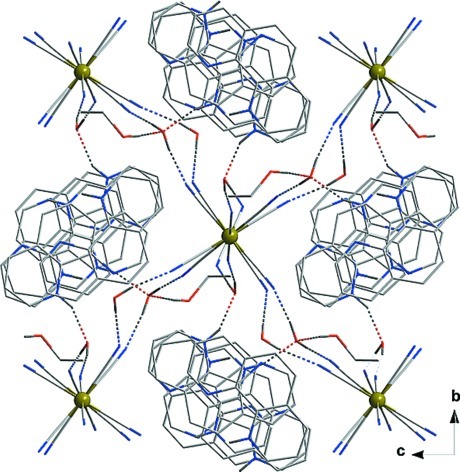
A three-dimensional supramolecular network of the title complex. Dotted lines represent hydrogen-bonding interactions.

## supplementary crystallographic information

### Comment

Design and synthesis of hexacyanometalate-based complexes have been widely studied because of their rich topologies and interesting properties (Tokoro & Ohkoshi, 2011). A large number of 3d-4f bimetallic systems have been synthesized by self-assembly of [*M*(CN)_6_]^3-^ (*M* = Fe, Cr, Co) building blocks and lanthanide ions in the presence of organic ligands (Andruh *et al.*, 2009). Recently, our group have aimed to prepare low-dimensional 3d-4f assembiles, employing [Fe(CN)_6_]^3-^ as aprecusor to react with lanthanide ions (Nd, Eu) and the chelating ligand 1,10-phenanthroline (phen). Unexceptedly, a new ion-pair complex, (Hphen)_3_.Fe(CN)_6_.CH_3_CH_2_OH.3H_2_O was obtained, in which the lanthanide ions were not involved.

The asymmetric unit of the title complex consists of two half [Fe(CN)_6_]^3-^ anions located on inversion centers, three protonated cations, [Hphen]^+^, an ethanol and three water molecules (Fig. 1). Both Fe atoms are in six-coordinated octahedral geometry. The average bond distances of Fe—C and C—N are 1.942 (6) and 1.154 (3) Å, respectively, while the Fe—C—N angles derivate slightly from the linearity with the angles spanning from 177.8 (2) to 179.7 (2)°. The crystal structure is stabilized by hydrogen-bonding interactions involving [Fe(CN)_6_]^3-^ units, [Hphen]^+^ cations, ethanol and water of hydration to form a three-dimensional supermolecular network (Fig. 2).

### Experimental

Single crystals of the title complex were prepared at room temperature by slow diffusion of an ethanol solution (3 ml) containing Ln(NO_3_)_3_.6H_2_O (Ln = Nd, Eu; 0.10 mmol) and phen (0.20 mmol) into a water solution (15 ml) of K_3_[Fe(CN)_6_].H_2_O (0.10 mmol). After about two weeks, block-shaped yellow crystals were obtained.

### Refinement

The C-bound H atoms were included in the refinement at calculated positions in riding mode (C—H = 0.95–0.99 Å, *U*_iso_(H) = 1.2 or 1.5 *U*_eq_(C)). The H atoms of water molecules and N-bound H atoms of the phen ligands were located from difference Fourier maps and refined in riding modes (O—H = 0.85 and N—H = 0.88 Å, *U*_iso_(H) = 1.5 *U*_eq_(O) or 1.2 *U*_eq_(N)). The hydroxyl H atom of the ethanol molecule was located from a Fourier map and was included as riding mode (O—H = 0.84 Å, *U*_iso_(H) = 1.2 *U*_eq_(O)).

### Crystal data

**Table:**

(C_12_H_9_N_2_)_3_[Fe(CN)_6_]·C_2_H_6_O·3H_2_O	*F*(000) = 1780
*M**_r_* = 855.72	*D*_x_ = 1.388 Mg m^−^^3^
Monoclinic, *P*2_1_/*c*	Mo *K*α radiation, λ = 0.71073 Å
Hall symbol: -P 2ybc	Cell parameters from 6253 reflections
*a* = 20.5744 (18) Å	θ = 2.5–27.4°
*b* = 14.8742 (13) Å	µ = 0.43 mm^−^^1^
*c* = 14.1594 (12) Å	*T* = 173 K
β = 109.123 (1)°	Block, colorless
*V* = 4094.1 (6) Å^3^	0.21 × 0.18 × 0.16 mm
*Z* = 4	

### Data collection

**Table:**

Bruker APEXII diffractometer	9412 independent reflections
Radiation source: fine-focus sealed tube	6179 reflections with *I* > 2σ(*I*)
Graphite monochromator	*R*_int_ = 0.049
φ & ω scans	θ_max_ = 27.5°, θ_min_ = 1.7°
Absorption correction: multi-scan (*SADABS*; Bruker, 2004)	*h* = −26→26
*T*_min_ = 0.915, *T*_max_ = 0.935	*k* = −19→18
35350 measured reflections	*l* = −18→18

### Refinement

**Table:**

Refinement on *F*^2^	Primary atom site location: structure-invariant direct methods
Least-squares matrix: full	Secondary atom site location: difference Fourier map
*R*[*F*^2^ > 2σ(*F*^2^)] = 0.042	Hydrogen site location: inferred from neighbouring sites
*wR*(*F*^2^) = 0.115	H-atom parameters constrained
*S* = 1.01	*w* = 1/[σ^2^(*F*_o_^2^) + (0.0508*P*)^2^ + 1.7012*P*] where *P* = (*F*_o_^2^ + 2*F*_c_^2^)/3
9412 reflections	(Δ/σ)_max_ = 0.001
555 parameters	Δρ_max_ = 0.42 e Å^−^^3^
0 restraints	Δρ_min_ = −0.46 e Å^−^^3^

### Special details

**Table:**

Geometry. All e.s.d.'s (except the e.s.d. in the dihedral angle between two l.s. planes) are estimated using the full covariance matrix. The cell e.s.d.'s are taken into account individually in the estimation of e.s.d.'s in distances, angles and torsion angles; correlations between e.s.d.'s in cell parameters are only used when they are defined by crystal symmetry. An approximate (isotropic) treatment of cell e.s.d.'s is used for estimating e.s.d.'s involving l.s. planes.
Refinement. Refinement of *F*^2^ against ALL reflections. The weighted *R*-factor *wR* and goodness of fit *S* are based on *F*^2^, conventional *R*-factors *R* are based on *F*, with *F* set to zero for negative *F*^2^. The threshold expression of *F*^2^ > σ(*F*^2^) is used only for calculating *R*-factors(gt) *etc*. and is not relevant to the choice of reflections for refinement. *R*-factors based on *F*^2^ are statistically about twice as large as those based on *F*, and *R*- factors based on ALL data will be even larger.

### Fractional atomic coordinates and isotropic or equivalent isotropic displacement parameters (Å^2^)

**Table:**

	*x*	*y*	*z*	*U*_iso_*/*U*_eq_	
Fe1	0.5000	0.5000	0.5000	0.01691 (10)	
O1	0.61077 (8)	0.68067 (10)	1.22601 (11)	0.0260 (4)	
H1WA	0.5871	0.6602	1.2604	0.039*	
H1WB	0.5975	0.7337	1.2064	0.039*	
N1	0.53010 (10)	0.62058 (14)	0.34081 (15)	0.0311 (5)	
C1	0.51810 (11)	0.57457 (15)	0.39874 (16)	0.0213 (5)	
Fe2	1.0000	0.5000	0.5000	0.01750 (11)	
O2	0.74379 (8)	0.17371 (11)	−0.01841 (13)	0.0350 (4)	
H2WA	0.7173	0.1443	0.0054	0.053*	
H2WB	0.7779	0.1410	−0.0175	0.053*	
N2	0.56126 (11)	0.65586 (14)	0.64662 (15)	0.0339 (5)	
C2	0.53866 (12)	0.59643 (15)	0.59344 (16)	0.0236 (5)	
O3	0.90143 (8)	0.68762 (10)	0.10957 (12)	0.0296 (4)	
H3WA	0.9130	0.6578	0.1638	0.044*	
H3WB	0.9316	0.7276	0.1129	0.044*	
N3	0.64249 (10)	0.40996 (14)	0.54417 (15)	0.0320 (5)	
C3	0.59010 (11)	0.44465 (15)	0.52866 (16)	0.0226 (5)	
O4	0.74538 (11)	0.80279 (16)	−0.1391 (2)	0.0725 (8)	
H4	0.7081	0.8116	−0.1855	0.087*	
N4	0.86099 (10)	0.42701 (15)	0.50654 (15)	0.0344 (5)	
C4	0.91260 (11)	0.45459 (15)	0.50366 (16)	0.0226 (5)	
N5	0.92963 (11)	0.60560 (14)	0.30400 (15)	0.0337 (5)	
C5	0.95502 (11)	0.56681 (16)	0.37726 (17)	0.0237 (5)	
N6	0.98584 (11)	0.66414 (14)	0.62580 (15)	0.0316 (5)	
C6	0.99123 (11)	0.60252 (15)	0.57895 (16)	0.0220 (5)	
N7	0.58878 (9)	0.59499 (12)	1.05172 (13)	0.0208 (4)	
H7A	0.5982	0.6111	1.1146	0.025*	
C7	0.57651 (11)	0.65885 (16)	0.98177 (17)	0.0248 (5)	
H7	0.5778	0.7203	1.0006	0.030*	
N8	0.61145 (10)	0.47211 (13)	1.20218 (14)	0.0250 (4)	
C8	0.56189 (11)	0.63637 (16)	0.88212 (17)	0.0273 (5)	
H8	0.5537	0.6821	0.8328	0.033*	
N9	0.89140 (9)	0.61601 (13)	−0.06545 (14)	0.0240 (4)	
H9A	0.8970	0.6263	−0.0021	0.029*	
C9	0.55926 (11)	0.54730 (17)	0.85518 (17)	0.0256 (5)	
H9	0.5487	0.5314	0.7868	0.031*	
N10	0.92307 (9)	0.48236 (13)	0.07413 (14)	0.0237 (4)	
C10	0.57223 (10)	0.47915 (15)	0.92884 (16)	0.0211 (5)	
N11	0.73512 (9)	0.38648 (14)	−0.10638 (15)	0.0286 (4)	
C11	0.57002 (11)	0.38495 (16)	0.90624 (17)	0.0249 (5)	
H11	0.5600	0.3659	0.8389	0.030*	
N12	0.76927 (9)	0.32388 (14)	0.08598 (15)	0.0284 (5)	
H12A	0.7607	0.2874	0.0344	0.034*	
C12	0.58206 (11)	0.32297 (16)	0.98008 (17)	0.0261 (5)	
H12	0.5806	0.2609	0.9637	0.031*	
C13	0.59700 (10)	0.34912 (15)	1.08258 (16)	0.0221 (5)	
C14	0.60826 (11)	0.28672 (16)	1.16144 (17)	0.0277 (5)	
H14	0.6071	0.2239	1.1486	0.033*	
C15	0.62086 (12)	0.31807 (16)	1.25668 (18)	0.0315 (6)	
H15	0.6290	0.2772	1.3109	0.038*	
C16	0.62168 (12)	0.41098 (16)	1.27358 (17)	0.0300 (5)	
H16	0.6301	0.4313	1.3401	0.036*	
C17	0.59953 (10)	0.44070 (15)	1.10768 (16)	0.0199 (5)	
C18	0.58720 (10)	0.50614 (15)	1.02921 (15)	0.0194 (4)	
C19	0.87499 (12)	0.68438 (17)	−0.12971 (19)	0.0311 (6)	
H19	0.8695	0.7429	−0.1065	0.037*	
C20	0.86585 (12)	0.67089 (18)	−0.23002 (19)	0.0345 (6)	
H20	0.8537	0.7197	−0.2757	0.041*	
C21	0.87446 (11)	0.58643 (18)	−0.26289 (18)	0.0318 (6)	
H21	0.8687	0.5768	−0.3315	0.038*	
C22	0.89188 (11)	0.51358 (16)	−0.19514 (17)	0.0260 (5)	
C23	0.90248 (12)	0.42396 (18)	−0.22367 (18)	0.0320 (6)	
H23	0.8981	0.4116	−0.2913	0.038*	
C24	0.91866 (12)	0.35646 (17)	−0.15582 (18)	0.0308 (6)	
H24	0.9256	0.2976	−0.1766	0.037*	
C25	0.92544 (11)	0.37243 (15)	−0.05304 (16)	0.0235 (5)	
C26	0.94197 (12)	0.30442 (16)	0.02035 (18)	0.0294 (5)	
H26	0.9479	0.2440	0.0030	0.035*	
C27	0.94935 (12)	0.32691 (16)	0.11723 (18)	0.0286 (5)	
H27	0.9610	0.2821	0.1679	0.034*	
C28	0.93975 (11)	0.41590 (16)	0.14109 (17)	0.0268 (5)	
H28	0.9454	0.4299	0.2088	0.032*	
C29	0.91672 (10)	0.46000 (15)	−0.02137 (16)	0.0201 (5)	
C30	0.89972 (10)	0.53096 (15)	−0.09443 (16)	0.0214 (5)	
C31	0.71790 (12)	0.41970 (18)	−0.19830 (18)	0.0332 (6)	
H31	0.7101	0.3785	−0.2521	0.040*	
C32	0.71043 (12)	0.51142 (19)	−0.2212 (2)	0.0372 (6)	
H32	0.6975	0.5311	−0.2887	0.045*	
C33	0.72208 (12)	0.57241 (18)	−0.1450 (2)	0.0362 (6)	
H33	0.7178	0.6351	−0.1587	0.043*	
C34	0.74048 (11)	0.54076 (17)	−0.04558 (19)	0.0298 (6)	
C35	0.75300 (12)	0.59971 (19)	0.0385 (2)	0.0403 (7)	
H35	0.7491	0.6628	0.0278	0.048*	
C36	0.77032 (12)	0.5672 (2)	0.1328 (2)	0.0410 (7)	
H36	0.7782	0.6077	0.1873	0.049*	
C37	0.77683 (12)	0.47259 (18)	0.15146 (19)	0.0332 (6)	
C38	0.79561 (13)	0.4349 (2)	0.2477 (2)	0.0451 (7)	
H38	0.8049	0.4732	0.3043	0.054*	
C39	0.80072 (14)	0.3435 (2)	0.2609 (2)	0.0482 (8)	
H39	0.8135	0.3183	0.3261	0.058*	
C40	0.78688 (13)	0.2887 (2)	0.17787 (19)	0.0398 (6)	
H40	0.7899	0.2253	0.1861	0.048*	
C41	0.76419 (10)	0.41360 (17)	0.06965 (18)	0.0268 (5)	
C42	0.74609 (10)	0.44752 (16)	−0.03118 (17)	0.0254 (5)	
C43	0.7239 (2)	0.8784 (3)	0.0029 (3)	0.0806 (12)	
H43A	0.6742	0.8733	−0.0309	0.121*	
H43B	0.7340	0.9349	0.0406	0.121*	
H43C	0.7399	0.8277	0.0489	0.121*	
C44	0.7595 (2)	0.8775 (3)	−0.0716 (3)	0.0795 (12)	
H44A	0.7473	0.9333	−0.1116	0.095*	
H44B	0.8096	0.8792	−0.0357	0.095*	

### Atomic displacement parameters (Å^2^)

**Table:**

	*U*^11^	*U*^22^	*U*^33^	*U*^12^	*U*^13^	*U*^23^
Fe1	0.0227 (2)	0.0143 (2)	0.0139 (2)	−0.00103 (17)	0.00619 (16)	−0.00050 (18)
O1	0.0348 (9)	0.0182 (8)	0.0268 (9)	0.0025 (7)	0.0125 (7)	0.0052 (7)
N1	0.0398 (11)	0.0297 (12)	0.0240 (11)	−0.0038 (9)	0.0110 (9)	0.0042 (9)
C1	0.0251 (11)	0.0205 (12)	0.0170 (11)	−0.0003 (9)	0.0052 (9)	−0.0022 (9)
Fe2	0.0213 (2)	0.0155 (2)	0.0160 (2)	0.00027 (17)	0.00647 (16)	0.00038 (18)
O2	0.0283 (9)	0.0290 (10)	0.0509 (11)	0.0027 (7)	0.0172 (8)	0.0052 (8)
N2	0.0521 (13)	0.0237 (12)	0.0236 (11)	−0.0071 (10)	0.0093 (9)	−0.0031 (9)
C2	0.0334 (12)	0.0205 (12)	0.0170 (11)	0.0002 (9)	0.0084 (9)	0.0042 (9)
O3	0.0388 (9)	0.0227 (9)	0.0267 (9)	−0.0063 (7)	0.0099 (7)	0.0001 (7)
N3	0.0315 (11)	0.0302 (12)	0.0354 (12)	0.0027 (9)	0.0125 (9)	0.0045 (9)
C3	0.0292 (12)	0.0207 (12)	0.0183 (11)	−0.0015 (9)	0.0084 (9)	0.0017 (9)
O4	0.0425 (13)	0.0613 (16)	0.0932 (19)	0.0131 (11)	−0.0054 (12)	−0.0321 (14)
N4	0.0324 (11)	0.0392 (13)	0.0321 (12)	−0.0054 (9)	0.0113 (9)	−0.0005 (10)
C4	0.0268 (11)	0.0223 (12)	0.0171 (11)	−0.0007 (9)	0.0052 (9)	−0.0011 (9)
N5	0.0418 (12)	0.0306 (12)	0.0253 (11)	0.0016 (9)	0.0063 (9)	0.0041 (9)
C5	0.0270 (11)	0.0221 (12)	0.0226 (12)	−0.0007 (9)	0.0088 (9)	−0.0007 (10)
N6	0.0442 (12)	0.0250 (12)	0.0277 (11)	0.0042 (9)	0.0148 (9)	−0.0003 (9)
C6	0.0253 (11)	0.0226 (12)	0.0180 (11)	0.0003 (9)	0.0071 (9)	0.0048 (9)
N7	0.0221 (9)	0.0219 (10)	0.0185 (9)	0.0013 (7)	0.0070 (7)	0.0020 (8)
C7	0.0255 (11)	0.0233 (12)	0.0273 (13)	0.0011 (9)	0.0109 (9)	0.0053 (10)
N8	0.0312 (10)	0.0250 (11)	0.0190 (10)	0.0011 (8)	0.0085 (8)	0.0020 (8)
C8	0.0294 (12)	0.0298 (14)	0.0250 (12)	0.0041 (10)	0.0120 (10)	0.0087 (10)
N9	0.0238 (9)	0.0255 (11)	0.0223 (10)	−0.0011 (8)	0.0068 (8)	0.0024 (8)
C9	0.0209 (11)	0.0386 (15)	0.0179 (12)	0.0009 (10)	0.0073 (9)	0.0044 (10)
N10	0.0224 (9)	0.0290 (12)	0.0196 (10)	−0.0015 (8)	0.0067 (7)	0.0001 (8)
C10	0.0173 (10)	0.0280 (13)	0.0188 (11)	0.0015 (8)	0.0070 (8)	0.0009 (9)
N11	0.0264 (10)	0.0310 (12)	0.0275 (11)	−0.0008 (8)	0.0076 (8)	0.0004 (9)
C11	0.0229 (11)	0.0304 (13)	0.0213 (12)	−0.0011 (9)	0.0072 (9)	−0.0047 (10)
N12	0.0257 (10)	0.0335 (12)	0.0246 (11)	0.0004 (8)	0.0063 (8)	0.0007 (9)
C12	0.0261 (11)	0.0231 (13)	0.0284 (13)	0.0004 (9)	0.0080 (10)	−0.0031 (10)
C13	0.0206 (10)	0.0217 (12)	0.0233 (12)	0.0006 (9)	0.0062 (9)	−0.0011 (10)
C14	0.0314 (12)	0.0219 (13)	0.0280 (13)	0.0017 (10)	0.0072 (10)	0.0027 (10)
C15	0.0403 (14)	0.0250 (14)	0.0288 (13)	0.0012 (10)	0.0108 (11)	0.0079 (11)
C16	0.0377 (13)	0.0316 (14)	0.0199 (12)	0.0012 (11)	0.0084 (10)	0.0029 (10)
C17	0.0182 (10)	0.0212 (12)	0.0194 (11)	0.0001 (8)	0.0048 (8)	−0.0003 (9)
C18	0.0167 (9)	0.0227 (12)	0.0198 (11)	0.0007 (9)	0.0073 (8)	0.0007 (10)
C19	0.0285 (12)	0.0263 (13)	0.0390 (15)	0.0001 (10)	0.0117 (11)	0.0077 (11)
C20	0.0314 (13)	0.0377 (16)	0.0336 (14)	0.0014 (11)	0.0094 (11)	0.0154 (12)
C21	0.0251 (12)	0.0462 (17)	0.0247 (13)	−0.0008 (11)	0.0092 (10)	0.0080 (12)
C22	0.0196 (10)	0.0371 (15)	0.0203 (11)	−0.0004 (9)	0.0052 (9)	0.0023 (10)
C23	0.0325 (13)	0.0422 (16)	0.0221 (13)	0.0014 (11)	0.0100 (10)	−0.0050 (11)
C24	0.0336 (13)	0.0297 (14)	0.0304 (14)	0.0030 (10)	0.0123 (11)	−0.0064 (11)
C25	0.0213 (11)	0.0251 (13)	0.0231 (12)	−0.0005 (9)	0.0059 (9)	−0.0007 (10)
C26	0.0296 (12)	0.0238 (13)	0.0347 (14)	0.0021 (10)	0.0105 (10)	−0.0008 (11)
C27	0.0310 (12)	0.0258 (13)	0.0289 (13)	0.0016 (10)	0.0095 (10)	0.0091 (11)
C28	0.0263 (11)	0.0329 (14)	0.0198 (12)	−0.0002 (10)	0.0059 (9)	0.0037 (10)
C29	0.0150 (9)	0.0252 (12)	0.0189 (11)	−0.0009 (8)	0.0040 (8)	0.0008 (9)
C30	0.0175 (10)	0.0228 (12)	0.0234 (12)	−0.0002 (8)	0.0060 (9)	0.0012 (9)
C31	0.0284 (12)	0.0415 (16)	0.0278 (14)	−0.0030 (11)	0.0067 (10)	0.0015 (12)
C32	0.0268 (12)	0.0450 (18)	0.0389 (15)	−0.0004 (11)	0.0094 (11)	0.0148 (13)
C33	0.0239 (12)	0.0306 (15)	0.0531 (17)	−0.0003 (10)	0.0110 (11)	0.0106 (13)
C34	0.0178 (11)	0.0293 (14)	0.0404 (15)	−0.0033 (9)	0.0070 (10)	−0.0004 (12)
C35	0.0251 (12)	0.0316 (15)	0.0607 (19)	−0.0029 (10)	0.0095 (13)	−0.0089 (14)
C36	0.0246 (12)	0.0455 (17)	0.0493 (18)	−0.0052 (11)	0.0074 (12)	−0.0209 (14)
C37	0.0209 (11)	0.0451 (16)	0.0320 (14)	−0.0035 (10)	0.0064 (10)	−0.0099 (12)
C38	0.0339 (14)	0.070 (2)	0.0295 (15)	−0.0006 (14)	0.0082 (11)	−0.0141 (15)
C39	0.0469 (16)	0.070 (2)	0.0255 (15)	0.0043 (15)	0.0094 (12)	0.0062 (15)
C40	0.0349 (14)	0.0510 (18)	0.0328 (15)	0.0052 (12)	0.0103 (11)	0.0091 (13)
C41	0.0159 (10)	0.0329 (14)	0.0303 (13)	−0.0012 (9)	0.0059 (9)	−0.0037 (11)
C42	0.0151 (10)	0.0305 (14)	0.0295 (13)	−0.0009 (9)	0.0056 (9)	0.0009 (10)
C43	0.069 (2)	0.093 (3)	0.067 (3)	0.024 (2)	0.005 (2)	−0.012 (2)
C44	0.070 (2)	0.063 (3)	0.107 (3)	−0.024 (2)	0.031 (2)	−0.037 (2)

### Geometric parameters (Å, º)

**Table:**

Fe1—C2	1.937 (2)	C13—C14	1.412 (3)
Fe1—C2^i^	1.937 (2)	C14—C15	1.369 (3)
Fe1—C1^i^	1.943 (2)	C14—H14	0.9500
Fe1—C1	1.943 (2)	C15—C16	1.402 (3)
Fe1—C3	1.946 (2)	C15—H15	0.9500
Fe1—C3^i^	1.946 (2)	C16—H16	0.9500
O1—H1WA	0.8500	C17—C18	1.436 (3)
O1—H1WB	0.8501	C19—C20	1.385 (4)
N1—C1	1.155 (3)	C19—H19	0.9500
Fe2—C6	1.934 (2)	C20—C21	1.371 (4)
Fe2—C6^ii^	1.934 (2)	C20—H20	0.9500
Fe2—C4^ii^	1.937 (2)	C21—C22	1.413 (3)
Fe2—C4	1.937 (2)	C21—H21	0.9500
Fe2—C5^ii^	1.952 (2)	C22—C30	1.406 (3)
Fe2—C5	1.952 (2)	C22—C23	1.430 (3)
O2—H2WA	0.8499	C23—C24	1.354 (3)
O2—H2WB	0.8503	C23—H23	0.9500
N2—C2	1.156 (3)	C24—C25	1.436 (3)
O3—H3WA	0.8502	C24—H24	0.9500
O3—H3WB	0.8503	C25—C29	1.408 (3)
N3—C3	1.149 (3)	C25—C26	1.410 (3)
O4—C44	1.432 (4)	C26—C27	1.371 (3)
O4—H4	0.8400	C26—H26	0.9500
N4—C4	1.151 (3)	C27—C28	1.396 (3)
N5—C5	1.152 (3)	C27—H27	0.9500
N6—C6	1.158 (3)	C28—H28	0.9500
N7—C7	1.335 (3)	C29—C30	1.439 (3)
N7—C18	1.357 (3)	C31—C32	1.399 (4)
N7—H7A	0.8800	C31—H31	0.9500
C7—C8	1.384 (3)	C32—C33	1.370 (4)
C7—H7	0.9500	C32—H32	0.9500
N8—C16	1.324 (3)	C33—C34	1.413 (4)
N8—C17	1.362 (3)	C33—H33	0.9500
C8—C9	1.375 (3)	C34—C42	1.401 (3)
C8—H8	0.9500	C34—C35	1.432 (4)
N9—C19	1.332 (3)	C35—C36	1.354 (4)
N9—C30	1.358 (3)	C35—H35	0.9500
N9—H9A	0.8800	C36—C37	1.429 (4)
C9—C10	1.416 (3)	C36—H36	0.9500
C9—H9	0.9500	C37—C38	1.406 (4)
N10—C28	1.334 (3)	C37—C41	1.408 (3)
N10—C29	1.357 (3)	C38—C39	1.372 (4)
C10—C18	1.410 (3)	C38—H38	0.9500
C10—C11	1.435 (3)	C39—C40	1.381 (4)
N11—C31	1.327 (3)	C39—H39	0.9500
N11—C42	1.361 (3)	C40—H40	0.9500
C11—C12	1.354 (3)	C41—C42	1.443 (3)
C11—H11	0.9500	C43—C44	1.468 (5)
N12—C40	1.337 (3)	C43—H43A	0.9800
N12—C41	1.352 (3)	C43—H43B	0.9800
N12—H12A	0.8800	C43—H43C	0.9800
C12—C13	1.435 (3)	C44—H44A	0.9900
C12—H12	0.9500	C44—H44B	0.9900
C13—C17	1.404 (3)		
			
C2—Fe1—C2^i^	180.0	N7—C18—C17	119.59 (19)
C2—Fe1—C1^i^	93.08 (9)	C10—C18—C17	120.8 (2)
C2^i^—Fe1—C1^i^	86.92 (9)	N9—C19—C20	120.6 (2)
C2—Fe1—C1	86.92 (9)	N9—C19—H19	119.7
C2^i^—Fe1—C1	93.08 (9)	C20—C19—H19	119.7
C1^i^—Fe1—C1	180.0	C21—C20—C19	119.4 (2)
C2—Fe1—C3	91.20 (9)	C21—C20—H20	120.3
C2^i^—Fe1—C3	88.80 (9)	C19—C20—H20	120.3
C1^i^—Fe1—C3	90.25 (9)	C20—C21—C22	120.3 (2)
C1—Fe1—C3	89.75 (9)	C20—C21—H21	119.9
C2—Fe1—C3^i^	88.80 (9)	C22—C21—H21	119.9
C2^i^—Fe1—C3^i^	91.20 (9)	C30—C22—C21	117.8 (2)
C1^i^—Fe1—C3^i^	89.75 (9)	C30—C22—C23	118.7 (2)
C1—Fe1—C3^i^	90.25 (9)	C21—C22—C23	123.4 (2)
C3—Fe1—C3^i^	180.0	C24—C23—C22	121.0 (2)
H1WA—O1—H1WB	109.7	C24—C23—H23	119.5
N1—C1—Fe1	177.9 (2)	C22—C23—H23	119.5
C6—Fe2—C6^ii^	180.0	C23—C24—C25	121.1 (2)
C6—Fe2—C4^ii^	90.21 (9)	C23—C24—H24	119.5
C6^ii^—Fe2—C4^ii^	89.79 (9)	C25—C24—H24	119.5
C6—Fe2—C4	89.79 (9)	C29—C25—C26	117.0 (2)
C6^ii^—Fe2—C4	90.21 (9)	C29—C25—C24	119.7 (2)
C4^ii^—Fe2—C4	180.0	C26—C25—C24	123.3 (2)
C6—Fe2—C5^ii^	89.63 (9)	C27—C26—C25	118.9 (2)
C6^ii^—Fe2—C5^ii^	90.37 (9)	C27—C26—H26	120.5
C4^ii^—Fe2—C5^ii^	91.60 (9)	C25—C26—H26	120.5
C4—Fe2—C5^ii^	88.40 (9)	C26—C27—C28	119.7 (2)
C6—Fe2—C5	90.37 (9)	C26—C27—H27	120.2
C6^ii^—Fe2—C5	89.63 (9)	C28—C27—H27	120.2
C4^ii^—Fe2—C5	88.40 (9)	N10—C28—C27	123.7 (2)
C4—Fe2—C5	91.60 (9)	N10—C28—H28	118.2
C5^ii^—Fe2—C5	180.0	C27—C28—H28	118.2
H2WA—O2—H2WB	109.4	N10—C29—C25	124.3 (2)
N2—C2—Fe1	177.8 (2)	N10—C29—C30	117.3 (2)
H3WA—O3—H3WB	109.4	C25—C29—C30	118.4 (2)
N3—C3—Fe1	178.2 (2)	N9—C30—C22	119.6 (2)
C44—O4—H4	109.5	N9—C30—C29	119.4 (2)
N4—C4—Fe2	179.3 (2)	C22—C30—C29	121.0 (2)
N5—C5—Fe2	178.5 (2)	N11—C31—C32	124.4 (2)
N6—C6—Fe2	179.7 (2)	N11—C31—H31	117.8
C7—N7—C18	122.30 (19)	C32—C31—H31	117.8
C7—N7—H7A	118.9	C33—C32—C31	119.0 (2)
C18—N7—H7A	118.9	C33—C32—H32	120.5
N7—C7—C8	120.6 (2)	C31—C32—H32	120.5
N7—C7—H7	119.7	C32—C33—C34	119.0 (2)
C8—C7—H7	119.7	C32—C33—H33	120.5
C16—N8—C17	116.6 (2)	C34—C33—H33	120.5
C9—C8—C7	119.4 (2)	C42—C34—C33	117.2 (2)
C9—C8—H8	120.3	C42—C34—C35	120.1 (2)
C7—C8—H8	120.3	C33—C34—C35	122.7 (3)
C19—N9—C30	122.2 (2)	C36—C35—C34	121.2 (3)
C19—N9—H9A	118.9	C36—C35—H35	119.4
C30—N9—H9A	118.9	C34—C35—H35	119.4
C8—C9—C10	120.3 (2)	C35—C36—C37	120.8 (3)
C8—C9—H9	119.8	C35—C36—H36	119.6
C10—C9—H9	119.8	C37—C36—H36	119.6
C28—N10—C29	116.4 (2)	C38—C37—C41	117.8 (3)
C18—C10—C9	117.7 (2)	C38—C37—C36	123.5 (3)
C18—C10—C11	118.9 (2)	C41—C37—C36	118.7 (2)
C9—C10—C11	123.4 (2)	C39—C38—C37	120.8 (3)
C31—N11—C42	116.2 (2)	C39—C38—H38	119.6
C12—C11—C10	120.5 (2)	C37—C38—H38	119.6
C12—C11—H11	119.7	C38—C39—C40	118.9 (3)
C10—C11—H11	119.7	C38—C39—H39	120.6
C40—N12—C41	122.2 (2)	C40—C39—H39	120.6
C40—N12—H12A	118.9	N12—C40—C39	120.8 (3)
C41—N12—H12A	118.9	N12—C40—H40	119.6
C11—C12—C13	121.4 (2)	C39—C40—H40	119.6
C11—C12—H12	119.3	N12—C41—C37	119.4 (2)
C13—C12—H12	119.3	N12—C41—C42	119.6 (2)
C17—C13—C14	117.0 (2)	C37—C41—C42	120.9 (2)
C17—C13—C12	119.8 (2)	N11—C42—C34	124.2 (2)
C14—C13—C12	123.2 (2)	N11—C42—C41	117.6 (2)
C15—C14—C13	119.0 (2)	C34—C42—C41	118.2 (2)
C15—C14—H14	120.5	C44—C43—H43A	109.5
C13—C14—H14	120.5	C44—C43—H43B	109.5
C14—C15—C16	119.5 (2)	H43A—C43—H43B	109.5
C14—C15—H15	120.3	C44—C43—H43C	109.5
C16—C15—H15	120.3	H43A—C43—H43C	109.5
N8—C16—C15	123.8 (2)	H43B—C43—H43C	109.5
N8—C16—H16	118.1	O4—C44—C43	116.6 (3)
C15—C16—H16	118.1	O4—C44—H44A	108.1
N8—C17—C13	124.1 (2)	C43—C44—H44A	108.1
N8—C17—C18	117.20 (19)	O4—C44—H44B	108.1
C13—C17—C18	118.6 (2)	C43—C44—H44B	108.1
N7—C18—C10	119.62 (19)	H44A—C44—H44B	107.3

Symmetry codes: (i) −*x*+1, −*y*+1, −*z*+1; (ii) −*x*+2, −*y*+1, −*z*+1.

### Hydrogen-bond geometry (Å, º)

**Table:**

*D*—H···*A*	*D*—H	H···*A*	*D*···*A*	*D*—H···*A*
O1—H1*WA*···N1^iii^	0.85	1.97	2.822 (2)	176
O1—H1*WB*···N2^iv^	0.85	1.89	2.732 (3)	172
O2—H2*WA*···N3^v^	0.85	1.97	2.809 (3)	169
O2—H2*WB*···N4^v^	0.85	1.92	2.761 (3)	171
O3—H3*WA*···N5	0.85	2.05	2.891 (3)	168
O3—H3*WB*···N6^vi^	0.85	1.93	2.769 (3)	168
N7—H7*A*···O1	0.88	1.83	2.681 (2)	161
N9—H9*A*···O3	0.88	1.80	2.643 (2)	159
N12—H12*A*···O2	0.88	1.84	2.635 (3)	150
O4—H4···O1^vii^	0.84	1.99	2.813 (3)	167

Symmetry codes: (iii) *x*, *y*, *z*+1; (iv) *x*, −*y*+3/2, *z*+1/2; (v) *x*, −*y*+1/2, *z*−1/2; (vi) *x*, −*y*+3/2, *z*−1/2; (vii) *x*, −*y*+3/2, *z*−3/2.
